# The Interference of Pre-Processing Software for the Numerical Simulation of Groundwater on the Cognition of Environmental Students: Model Mesh Construction as an Example

**DOI:** 10.3390/ijerph20021203

**Published:** 2023-01-10

**Authors:** Guanru Zhang, Peng Lu, Yi Huang

**Affiliations:** 1 College of Ecology and Environment, Chengdu University of Technology, Chengdu 610059, China; 2 Department of Earth and Atmospheric Sciences, Indiana University, Bloomington, IN 47405, USA

**Keywords:** pre-processing software, cognition, education, interference

## Abstract

(1) Background: Software for the numerical simulation of groundwater plays an important role in studying environmental problems. However, it is still unclear whether the pre-processing software of the numerical simulation of groundwater has a negative effect on the cognition of undergraduates in the environmental field who only have basic groundwater flow and solute transport knowledge and software operation skills. (2) Methods: To explore this issue, we used software meshing as an example and selected undergraduates in the environmental field to conduct the questionnaire surveys. A total of 345 undergraduate answer sheets were received, and data analysis was carried out. The students were divided into two groups, one with and another without certain basic groundwater flow and solute transport knowledge or software operation skills. (3) Results: For undergraduate students with some basic knowledge or software operation ability, the proportion of students whose cognition was adversely interfered with by the pre-processing software was 64.3%, and the ratio of students not interfered with was 35.7%. For undergraduates without groundwater flow and solute transport knowledge and relevant software operation skills, the ratios were 63.2% and 36.8%, respectively. (4) Conclusions: Pre-processing software numerical simulation of groundwater could negatively interfere with students’ cognition. The basic groundwater flow and solute transport knowledge and software operation skills did not observably reduce the interference degree (*p* = 0.259) but had significant influences on the undergraduates’ thinking modes on the numerical simulation problems (*p* = 0.009). The interference was mainly caused by the significant difference between the level of knowledge possessed by the students and that represented by the pre-processing software. This paper provides basic scientific data for the optimization of students’ knowledge structures and the improvement of teaching methods.

## 1. Introduction

Software for numerical simulation of groundwater plays an important role in studying and solving various environmental problems [1,2,3,4,5,6,7]. This type of software generally includes three modules: pre-processing, numerical calculation, and post-processing. The three modules can also be independent codes. The pre-processing software/module is often used to generate complex grids with cells of different geometric shapes; it can also assist the numerical calculation module to complete the input of physical and chemical parameters and the setting of boundary conditions [8]. The pre-treatment process is the foundation of groundwater flow and solute transport numerical simulation, which determines whether the simulated result can accurately describe the actual environmental pollution processes. Undergraduates in the environmental field generally are only required to have some basic groundwater flow and solute transport knowledge and numerical simulation skills [9] and are not required to be proficient in both the theory and the software. Will pre-processing software developed by senior experts significantly and negatively interfere with the cognition of students new to the numerical simulation of groundwater? The answer to this question will help to better optimize teaching methods and students’ knowledge structures and improve the quality of enterprises and governments in dealing with actual environmental pollution problems.

Recently, numerous studies have been conducted on the role of various methods and tools in teaching software and modeling and how software can improve the quality of theoretical education. For the teaching of software and modeling, different teaching methods and tools have been developed to improve students’ spatial perception, promote the integration of theory and practical application, and facilitate the in-depth understanding of simulation processes, etc. For example, Huang, et al. [10] combined tangible 3D materials and a cognitive-apprenticeship strategy in their 3D modeling course. This method stimulated more meta-cognition behaviors in students, helped students to build knowledge and skills through gradually adding layers of simple information, and eventually allowed students to apply knowledge and skills more effectively to the problem-solving process. Deng [11] introduced virtual display technology in computer software courses, which improved students’ understanding of the knowledge in the textbooks and actual operation process. Li and Davis [12] introduced a sand tank in groundwater teaching to help students understand groundwater modeling and calibration. The results indicate that the sand tank is a good visualization tool, which enhanced the comprehension of groundwater flow nets and groundwater modeling based on finite differences. Gómez-Hernández [13] found that spreadsheets can help students understand the mechanics of building a model and thus enhance their learning experience for students facing the numerical solution of groundwater flow partial differential equations for the first time. Generally, new teaching methods and tools in software and simulation teaching can help students step-by-step more deeply grasp the knowledge in books and the operation process of software and modeling and can improve students’ ability to apply the knowledge and skills they have learned to handle practical problems.

Software, as a teaching tool for theoretical education, has been widely used in teaching hydrology, solute transport, mathematics, etc. For example, Aghakouchak and Habib [14] developed a hands-on modeling tool to facilitate students’ learning of the fundamentals of hydrologic processes and the basic concepts of model calibration and sensitivity analysis and to stimulate conceptual thinking. The teaching evaluations indicated that this software can significantly stimulate students’ interest and improve students’ understanding of complex and multi-faceted hydrological concepts and processes. Coelho and Sierakowski [15] developed a teaching software, PSOLeT, to support the teaching of particle swarm optimization fundamentals. This software can significantly advance students’ level of understanding of intelligent systems and their level of excitement. Çokça and Aktaş [16] added MATLAB programming to their courses and asked students to write their MATLAB codes based on the analytical solution of the two-dimensional pollution migration problem to improve students’ understanding of the solute transport process. The results indicated that MATLAB programming can enhance the understanding of contaminate transport in soil and the role of different parameters in transport. Zengin [17] combined mathematics software and a flipped classroom approach in double integrals teaching. Classroom tests and questionnaires showed that this combined teaching method can make lessons more visual and enhance students’ understanding and retention of mathematical concepts. In general, software as a teaching tool can help students understand complex concepts and processes, improve students’ interest in learning and enthusiasm for their major, and provide practical skills for their future careers [18,19,20,21,22,23].

Although numerous studies have been conducted on the positive effects of various methods and tools on software teaching or the effects of software on improving teaching quality, to date little attention has been paid to the negative interference of numerical simulation software or its pre-processing module/software on students’ cognition. In this study, we focused on the interference of groundwater numerical simulation pre-processing software on students’ cognition and hypothesized that the pre-processing software might have a negative interference with the cognition of environmental undergraduate students who are just getting started in the numerical simulation of groundwater. To evaluate these negative effects, we used meshing as an example and selected undergraduates from the College of Ecology and Environment, Chengdu University of Technology, China, for questionnaire assessment. Based on the questionnaire data, the interference was quantified and discussed.

## 2. Materials and Methods

### 2.1. Question Design

Twelve questions were designed to investigate the interference of pre-processing software of groundwater numerical simulation on students’ cognition (Supplementary Materials). The twelve questions involved four types (Table 1): (1) Basic knowledge of groundwater flow and solute transport. This included four sub-questions: the basic concept of groundwater, Darcy’s law, the solute convection process, and the solute hydrodynamic dispersion process. According to the level of students’ knowledge, three levels and scores from low to high were assigned for each sub-question. These questions were used to determine the students’ levels of groundwater flow knowledge. (2) Operational skills in numerical simulation software for groundwater flow and solute transport, which included four sub-questions involving the setting of initial and boundary conditions, grid meshing, calculation result export, and a numerical simulation calculation method. According to the level of students’ skills, three levels and scores from low to high were assigned for each sub-question. These questions were used to evaluate the students’ software operation skills. (3) Choice of meshing and the reason for the choice. The questions were “Using a groundwater numerical simulation software based on the finite volume method to simulate the transport of pollutants released by point pollution sources in the aquifer. What is the most appropriate meshing?” and “The reason for the meshing selection in Question 3.1?”. For the former question, we provided a sketch of the problem scenario and three meshing schemes usually used by the pro-processing software, and for the latter, five potential reasons were provided. The answers to these two sub-questions were assigned scores 1–5 to facilitate the statistical analysis. Such questions were used to determine whether the students’ cognition had been interfered with and where the interference came from. (4) The importance of groundwater numerical simulation in the environmental field. The questions were “How important is the groundwater numerical simulation software in the theoretical research of environmental pollution?” and “How important is the groundwater numerical simulation software in environmental remediation engineering?”. According to importance, three levels and scores were set from low to high for each sub-question. These questions were used to assist in exploring the importance of optimization for the students’ basic knowledge and teaching methods.

### 2.2. Interference Criterion

Question 3.1, choice of meshing for a given scenario, was the key question to determine whether the students’ cognition had been interfered with. In Question 3.1, it was clarified that the simulation software uses the finite volume method. The idea of the finite volume method is to divide the study area into multiple control volumes and then calculate the flux passing through the boundary of the control volume and the mass or energy balance for each control volume, and the average value of the target variable in the control volume at the interested time is finally obtained [24,25,26]. The meshes of the finite volume method can adopt a more flexible geometric shape that better matches the terrain, rather than being limited to rectangles [27,28]. However, for calculation accuracy, the finite volume method still has strict requirements on the geometric shape of the grid: the connection line between the two control volume centers should be perpendicular to the common surface; the intersection point should coincide with the appropriate average position on the common surface [29].

The rectangular grid of Answer A in Question 3.1 met the geometric requirements of the finite volume method, and minor errors from the terrain matching were acceptable. The warped rectangular grid in Answer B could not meet the geometric requirements of the finite volume method, although it matched the terrain very well. For Answer C, the anisotropic permeability is common for porous media [30]. Although satisfying the geometric requirements of the finite volume method and matching the terrain, the Voronoi mesh might not yield an accurate flux for the vertical profile of the porous media with anisotropic permeability [29].

In summary, a rectangular grid (Answer A) was the best choice in this study in terms of spatial discretization and computational accuracy. If the pre-processing software only provides one best meshing scheme, the students will not be adversely induced; however, in practice, it generally provides a variety of meshing schemes, and the ultimate choice is up to the students/users. Multiple options mean a potential for induction and interference. For example, at a three-way intersection in a maze, the existence of two wrong bifurcations means potential interference, pointing to the unfavorable directions; if there are no bifurcations, there is no interference. Hence, Answers B and C in Questions 3.1 roughly represented the induction and negative interference of pre-processing software on the students’ cognition and were used as the criterion in this study.

### 2.3. Participants and Data Acquisition

The subjects of this study were freshman, sophomore, junior, and senior undergraduates at the College of Ecology and Environment, Chengdu University of Technology, China. Undergraduate majors included environmental science and engineering, environmental engineering, and environmental ecological engineering. A total of 345 answer sheets were received.

Questions were posted on the website (www.wjx.cn, accessed on 12 October 2022). A QR code was generated and sent through the mobile QQ app, and the teachers and the teaching assistants explained and guided the students in advance to ensure that most students could access the questionnaire and answer it. The questionnaire was conducted anonymously, and the answers were finally summarized into an Excel^©^ data table for subsequent analysis.

### 2.4. Data Analysis

We divided the students into two groups, A and B, for analysis (Table 2). If the students had some basic groundwater flow and solute transport knowledge or software operation ability, they were classified into Group A; otherwise, they were classified into Group B. The comparison of the two groups was used to analyze whether some relevant background knowledge could reduce the interference of pre-processing software.

IBM SPSS software (v26, SPSS Inc., Chicago, IL, USA) was employed for the statistical analysis of data. Mean and standard deviation were used to describe the score statistics succinctly. The Kolmogorov–Smirnov test was adopted for checking the normality; the Kruskal–Wallis test was used to assess the differences between Group A and Group B; *p* < 0.01 was used as the threshold of statistical significance. Box plots were employed to describe the probability distribution of the scores. The choice of different answers was analyzed in depth using a frequency histogram.

The sample numbers of Groups A and B were 182 and 163 and accounted for 52.8% and 47.2% of the total number of students, respectively (Table 2). Comparing Groups A and B, the scores of type 1, type 2, and union of type 1 and 2 questions had statistically significant differences (*p* = 0.000), and the mean scores of Group A were much higher than those of Group B, which indicated that the classification of students based on groundwater flow and solute transport knowledge or software skill was successful.

## 3. Results

**Figure 1:**
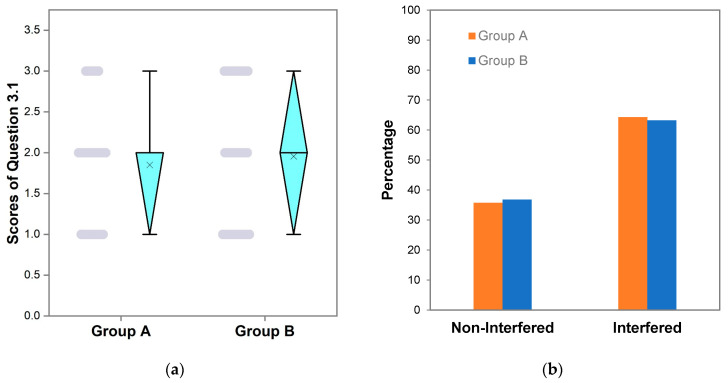
(**a**) Box plot and scatter diagram for Question 3.1 (gray dots represent discrete points of scores and × in the box plot represents mean); (**b**) distribution of the proportion of students whose cognition was interfered with by the pre-processing software.

Answering Question 3.1, i.e., the choice of meshing for a given scenario, correctly meant that the students’ cognition was not interfered with by the pre-processing software; otherwise, they were negatively interfered with. The mean score of Group A for Question 3.1 was 1.9 ± 0.7, almost the same as that (2.0 ± 0.8) of Group B (Table 3). *p* = 0.259 indicates that there were no significant differences between the scores of Groups A and B for Question 3.1. Figure 1a shows that the median values of scores of Question 3.1 for Groups A and B were both 2.0, higher than the mean values, which indicates that both scores exhibited a left-skewed distribution, i.e., most students tended to choose either B or C as the answer. This shows that the proportion of students whose cognitions were negatively interfered with by the pre-processing software was relatively high.

To quantitatively analyze the interference in-depth, we plotted the percentage of students who had been interfered with by the pre-processing software in Groups A and B (Figure 1b). The number of interfered undergraduates accounted for 64.3% of the total number of undergraduates in Group A, and the proportion of those not interfered with was 35.7%. The former was 1.8 times that of the latter. In Group B, the proportion of undergraduate students who had been interfered with was 63.2%, and the proportion of those who had not been interfered with was 36.8%. The former was 1.7 times the latter. In Group A and B, the number of students whose cognitions had been negatively interfered with by the pre-processing software was much higher than that of those non-interfered with.

Because there were no statistically significant differences in the scores of Question 3.1 for Group A and B (*p* = 0.259), compared with Group B, the proportion of undergraduates who had been interfered with in Group A only increased slightly from 63.2% to 64.3% (Figure 1b). This means that basic groundwater flow and solute transport knowledge and software skills did not significantly reduce the interference of pre-processing software on cognition.

In general, pre-processing software can negatively interfere with students’ cognition. Basic groundwater flow and solute transport knowledge and software skills had almost no effect on reducing the degree of interference.

## 4. Discussion

### 4.1. Interference Source

**Figure 2:**
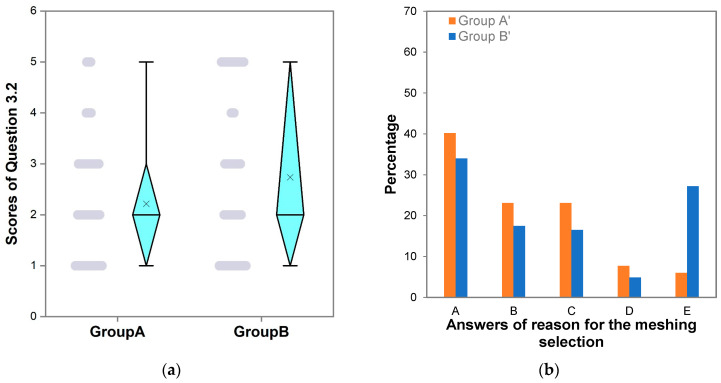
(**a**) Box plot and scatter diagram for Question 3.2 (gray dots represent discrete points of scores and × in the box plot represents mean); (**b**) for different answers to Question 3.2, the distribution of the percentage of students interfered with by pre-processing software.

We investigated the interference source by analyzing students’ answers to Question 3.2, the reason for the meshing selection. The mean for the Group A scores was 2.2 ± 1.1, smaller than that of Group B (2.7 ± 1.6) (Table 3). *p* = 0.009 indicates that the scores (answers) of Question 3.2 for Groups A and B had statistically significant differences. Figure 2a indicates that the median values of the scores of Question 3.2 were both 2.0, less than the means, indicating that both the scores of the two groups exhibited right-skewed distribution, i.e., more than half of the students in each group chose answers A and B.

To analyze the interference sources in depth, we plotted the percentage distribution of students interfered with by pre-processing software for answers to Question 3.2 (Figure 2b). The students who had been interfered with by the pre-processing software in Group A were classified as Group A’; those who had been interfered with in Group B were assigned as Group B’. The total number of undergraduates in Group A’ was 117, and the total number of undergraduates in Group B’ was 103.

For Question 3.2, the answers A “Matches the terrain” and D “Large and luxurious in the form” represented that the interference came from the visual effects. In Group A’, undergraduate students who answered A and D accounted for 47.9% of the total number of undergraduates in Group A’ (Figure 2b); the proportion was lower in Group B’ (38.9%). The learning of basic groundwater flow and solute transport knowledge and software operation had some effects on increasing visual interference by 9%.

Answer B, “It is easier for the post-processing of spatial data”, to Question 3.2 represented that the interference was sourced from data post-processing. In Group A’, the undergraduate students who answered B accounted for 23.1% of the total undergraduate students in Group A’ (Figure 2b), while the percentage was 17.5% in Group B’. The basic groundwater flow and solute transport knowledge and software operation skills somewhat increased the interference from this source by 5.6%.

Answer C, “Meets the requirements of numerical calculation methods”, to Question 3.2 represented that the interference came from the partial understanding of the numerical calculation methods. In Group A’, undergraduates who answered C accounted for 23.1%; the proportion was 16.5% in Group B’ (Figure 2b). Bearing basic groundwater flow and solute transport knowledge and software operation skills would urge the students to consider the problem from the essential process of numerical computation. However, because the relevant knowledge of undergraduates was not profound, they were still misled by the pre-processing software. The percentage of students who had been interfered with increased by 6.6%.

Answer E, “Others”, to Question 3.2 represented other potential interference sources. In Group A’, this interference source had the lowest proportions compared to the aforementioned three sources (Figure 2b). However, in Group B’, this source was the second largest source of interference.

In summary, although basic groundwater flow and solute transport knowledge and software skills had almost no effect on reducing the degree of interference of pre-processing software, they significantly affected how students think about problems.

### 4.2. Limited Prior Knowledge and Requirement for Teaching Improvements

All of the above interference sources are likely related to students’ limited prior knowledge. When the questions are raised, students will use their prior knowledge to extract relevant information from the questions and then add information from their prior knowledge to make different choices [31]. Students’ knowledge is fragmented, lacks connection [32], and does not cover all required domains. The pre-processing software was jointly developed by experts in multiple fields, representing a deeper and more comprehensive professional knowledge. The knowledge possessed/represented by students and that in the pre-processing software were not at the same level. Therefore, the students tended to consider and solve the problem based on their limited prior knowledge of the aspects of the visual effects, data post-processing, and numerical simulation, thus being induced and negatively interfered with by the pre-processing software.

One interesting phenomenon was that the answers to Question 3.1 from Groups A and B showed only negligible differences (64.3% vs. 63.2%). This probably implies that the difference between the level of knowledge possessed by the undergraduates and that represented by the pre-processing software was huge. The limited basic knowledge of undergraduates was not enough to remove the interference exerted by wrong options. This is like a beaker with 10 mol NaCl; adding 1 mol NaCl to it has little effect on the total amount.

**Figure 3:**
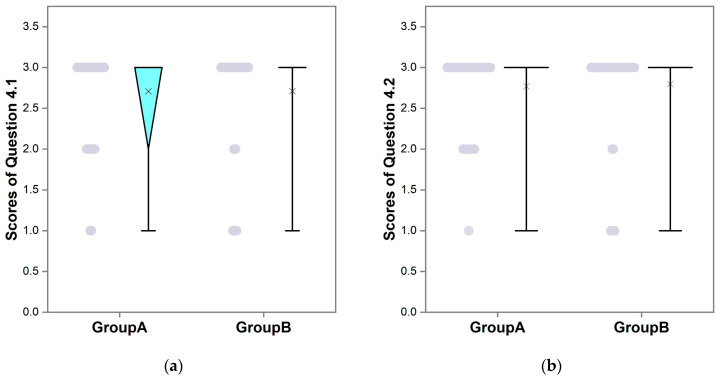
Box plot and scatter diagram for Questions 4.1 (**a**) and 4.2 (**b**). Gray dots represent discrete points of scores and × in the box plot represents mean.

Students’ awareness of the importance of numerical simulation of groundwater in theoretical and applied research can be evaluated by Questions 4.1 and 4.2 (Table 4). The means of the scores from Groups A and B were all larger than 2.7, close to the maximum value (3.0). *p* = 0.167 for Question 4.1 and *p* = 0.036 for Question 4.2 suggest that there was no statistically significant difference for the answers to the two questions from the Group A and B students. Figure 3 shows that for Questions 4.1 and 4.2, both the means for Groups A and B were smaller than medians, indicating that the scores exhibited a left-skewed distribution for both cases, i.e., most students selected C as the answer. In summary, most students in both Groups A and B believed that numerical simulation was important to study environmental pollution and prevention problems.

Although students have realized that simulation software plays a critical role in theoretical and applied research in the field of the environment, how to reconcile the inconsistency of the knowledge level between environmental students and the pre-processing software is still an unsolved problem in undergraduate education in universities. This highlights the new requirements to optimize the knowledge structure of undergraduates in the environmental field and to improve the relevant teaching methods and tools.

### 4.3. Limitations

Two potential explanations exist for the results of the meshing choices. Analyzing from the software perspective as we did in this study, incorrect mesh selection represented possible interference. Analyzing from the perspective of students, the choice of different grids reflects the level of knowledge that students have mastered, which can be used to determine whether students have received enough education to distinguish between different choices correctly. However, no matter from which angle, the fact that the dominant number of students made the wrong grid selections means that a significant difference existed between the knowledge students had mastered and the knowledge represented by the software. This difference can induce errors in questionnaires/exams mesh selection results and may cause unfavorable engineering results in practical applications.

The ultimate goal of this article was to discover problems in teaching and provide observations to optimize students’ knowledge structure and teaching methods, and not to prove which angle of explanation is more reasonable and correct. No matter from which points of view, the final results were approximately the same; both interpretations could support the ultimate educational goal of this research well.

## 5. Conclusions

To explore whether the pre-processing software of the numerical simulation of groundwater negatively affected the cognition of undergraduates in the environmental field who only have basic groundwater flow and solute transport knowledge and software operation skills, this study used meshing as an example and selected undergraduates in the environmental field to conduct the questionnaire surveys. Through the analysis of 345 questionnaires, the following conclusions were drawn: (1) >~63% of undergraduates’ cognition was adversely interfered with by pre-processing software for numerical simulations of groundwater; (2) basic groundwater flow and solute transport knowledge and numerical simulation skills did not have a significant effect on the level of interference, but they could significantly affect the way that students thought about numerical simulation problems; (3) the interference was mainly caused by the significant difference between the level of knowledge possessed by the undergraduates and that represented by the pre-processing software. This study provides basic scientific data to optimize the knowledge structure of undergraduates in the environmental field and to improve the relevant teaching methods and tools.

## Figures and Tables

**Table 1:** Questionnaire design.

Question Types	Descriptions	Number of Sub-Questions	Score Setting	Comments
Type 1	Basic knowledge of groundwater flow and solute transport	4	Answers A–C were scored 1–3, respectively	The higher the score, the better the professional knowledge
Type 2	Operational skills in numerical simulation software of groundwater	4	Answers A–C were scored 1–3, respectively	The higher the score, the better the software operation skills
Type 3	Choice of meshing and the reason	2	Answers A–E were scored 1–5, respectively	Scores only represented specific choices.
Type 4	Importance of numerical simulation of groundwater in the environmental field	2	Answers A–C were scored 1–3, respectively	The higher the score, the more important the numeral simulation.

**Table 2:** Statistics of Groups A and B scores.

Question Types	Group A (*n* = 182, 52.8%) ^2^			Group B (*n* = 163, 47.2%)			K-W Test ^4^
	Mean	Standard Deviation	Normality Test ^3^	Mean	Standard Deviation	Normality Test	*p*-Value
Type 1 and 2 ^1^	13.0	3.6	0.000	8.0	0.0	0.000	0.000
Type 1	7.2	2.0	0.000	4.0	0.0	0.000	0.000
Type 2	5.8	2.1	0.000	4.0	0.0	0.000	0.000
Type 3	4.1	1.3	0.000	4.7	1.8	0.000	0.002
Type 4	5.5	0.9	0.000	5.5	1.2	0.000	0.070

^1^ Union of type 1 and 2 questions. ^2^ The sample numbers of Group A and B were 182 and 163 and accounted for 52.8% and 47.2% of the total number of students, respectively. ^3^ The Kolmogorov–Smirnov test was used to check normality. ^4^ Kruskal–Wallis test.

**Table 3:** Statistics of scores of type-3 questions.

Sub-Questions	Group A			Group B			K-W Test
	Mean	Standard Deviation	Normality Test	Mean	Standard Deviation	Normality Test	*p*-Value
3.1 Choice of meshing for a given scenario.	1.9	0.7	0.000	2.0	0.8	0.000	0.259
3.2 The reason for the meshing selection.	2.2	1.1	0.000	2.7	1.6	0.000	0.009

**Table 4:** Statistics of scores of type-4 questions.

Sub-Questions	Group A			Group B			K-W Test
	Mean	Standard Deviation	Normality Test	Mean	Standard Deviation	Normality Test	*p*-Value
4.1 The importance of numerical simulation in theoretical research	2.7	0.5	0.000	2.7	0.7	0.000	0.167
4.2 The importance of numerical simulation in environmental remediation engineering	2.8	1.4	0.000	2.8	0.6	0.000	0.036

## Data Availability

The data presented in this study are available from the corresponding author on reasonable request.

## Supplementary Materials

The following supporting information can be downloaded at: https://www.mdpi.com/article/10.3390/ijerph20021203/s1, Questionnaire.
