# Supplementary material for: The Interference of Pre-Processing Software for the Numerical Simulation of Groundwater on the Cognition of Environmental Students: Model Mesh Construction as an Example
Source: Int J Environ Res Public Health. 2023 Jan 10;20(2):1203. doi: 10.3390/ijerph20021203 (PMC9859239; doi:10.3390/ijerph20021203)
Supplement: Supplementary file 1 [file ijerph-20-01203-s001.zip › ijerph-2079338-supplementary.pdf]

Questionnaire

1. Basic knowledge of groundwater flow and solute transport

1.1 How well do you understand the concepts of saturated zone, unsaturated zone, aquifer, etc.? (   )

A. Never learned.

B. With limited knowledge.

C. Proficient

1.2 How well do you master Darcy's Law? (   )

A. Never learned.

B. With limited knowledge.

C. Proficient

1.3 How well do you master the concept and theoretical formula of the advection process of solute transport? (   )

A. Never learned.

B. With limited knowledge.

C. Proficient

1.4 How well do you master the concept and theoretical formula of the hydrodynamic dispersion of solute transport? (   )

A. Never learned.

B. With limited knowledge.

C. Proficient

2. Operational skills of numerical simulation software of groundwater.

2.1 Can you skillfully use numerical simulation software to set initial and boundary conditions? (   )

A. Never learned.

B. With limited knowledge.

C. Proficient

2.2 Can you proficiently make gridding in numerical simulation software for a study area? (   )

A. Never learned.

B. With limited knowledge.

C. Proficient

2.3 Can you proficiently export the calculation results (graphs or datasets) from numerical simulation software? (   )

A. Never learned.

B. With limited knowledge.

C. Proficient

2.4. What is your level of the knowledge on numerical simulation methods such as finite difference, finite element, and finite volume? (   )

A. Never learned.

B. With limited knowledge.

C. Proficient

3. Meshing for numerical simulation

3.1. Using a groundwater numerical simulation software based on the finite volume method to simulate the transport of pollutants released by point pollution sources in the aquifer (see the figure below). What is the most appropriate meshing? (   )

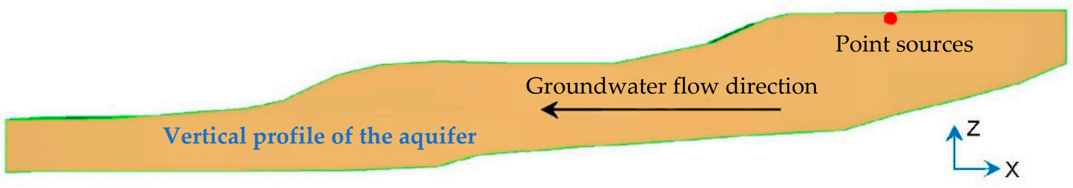

A.

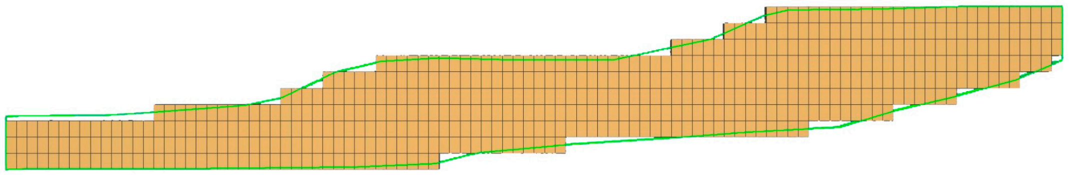

B.

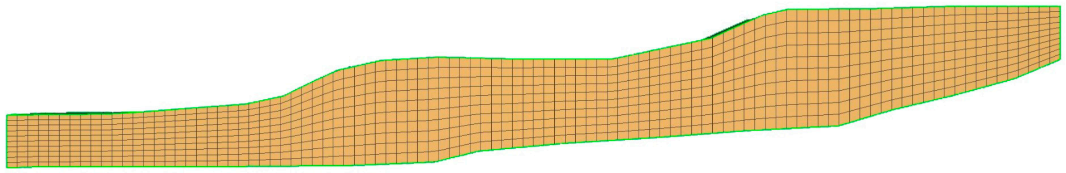

C.

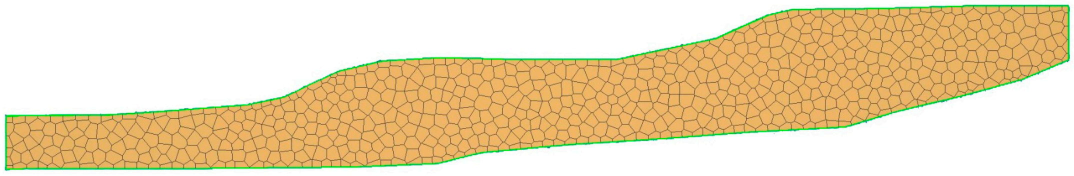

3.2. The reason for the meshing selection in Question 3.1? (   )

A. Matches the terrain

B. It is easier for the post-processing of spatial data

C. Meets the requirements of numerical calculation methods

D. “Large and luxurious” in the form

E. Others

4. The importance of groundwater numerical simulation in the field of environmental pollution and prevention

4.1 How important is the groundwater numerical simulation software in the theoretical research of environmental pollution? (   )

A. Insignificant

B. modest

C. Important

4.2 How important is the groundwater numerical simulation software in environmental remediation engineering? (   )

A. Insignificant

B. modest

C. Important
